# The clinical implications of adult-onset henoch-schonelin purpura

**DOI:** 10.1186/1476-7961-9-9

**Published:** 2011-05-27

**Authors:** Warit Jithpratuck, Yasmin Elshenawy, Hana Saleh, George Youngberg, David S Chi, Guha Krishnaswamy

**Affiliations:** 1 Departments of Internal Medicine, Quillen College of Medicine, East Tennessee State University, TN, USA; 2 Department of Pathology, Quillen College of Medicine, East Tennessee State University, TN, USA; 3 The James H. Quillen VA Medical Center, Johnson City, TN, USA

## Abstract

Henoch-Schonlein Purpura (HSP) is a small vessel vasculitis mediated by IgA-immune complex deposition. It is characterized by the clinical tetrad of non-thrombocytopenic palpable purpura, abdominal pain, arthritis and renal involvement. Pathologically, it can be considered a form of immune complex-mediated leukocytoclastic vasculitis (LCV) involving the skin and other organs. Though it primarily affects children (over 90% of cases), the occurrence in adults has been rarely reported. Management often involves the use of immunomodulatory or immune-suppressive regimens.

## Introduction

Henoch-Schonlein Purpura (HSP) is a small vessel vasculitis mediated by IgA-immune complex deposition. It is characterized by the clinical tetrad of non-thrombocytopenic palpable purpura, abdominal pain, arthritis and renal involvement [1]. Pathologically, it can be considered a form of leukocytoclastic vasculitis that can involve not only the skin but other tissues as well. Though it primarily affects children (over 90% of cases), the occurrence in adults has been rarely reported (3.4 to 14.3 cases per million). This low incidence could be due to either under-diagnosis or misdiagnosis.

Typically the disorder is commoner in males and may follow an infectious illness [2]. In the cases reported in children, the majority (of over 75%) of cases presented with an eruption, while up to 66% presented with abdominal pain and close to 50% the cases demonstrated renal involvement [2]. In children, the disorder is often self-limiting, while a more complicated course has been described in adults, including a high incidence of renal insufficiency developing in almost 50% of those patients who showed renal involvement. It appears that patients over 20 years old with bloody stools, relapsing disease and persistent eruption are more likely to progress onto complications [3]. Besides renal disease, cardiac, pulmonary, ocular, gastrointestinal and neurological complications have been described in this disorder. In that sense, this is truly a multisystem disease and may result in considerable morbidity and mortality in some patients. A variety of disorders have been associated with HSP including infection with Helicobacter pylori, hepatitis B and certain malignancies. The following review describes the course, complications and management of adult-onset HSP.

### Representative Case Studies

#### Case 1

**Figure 1:**
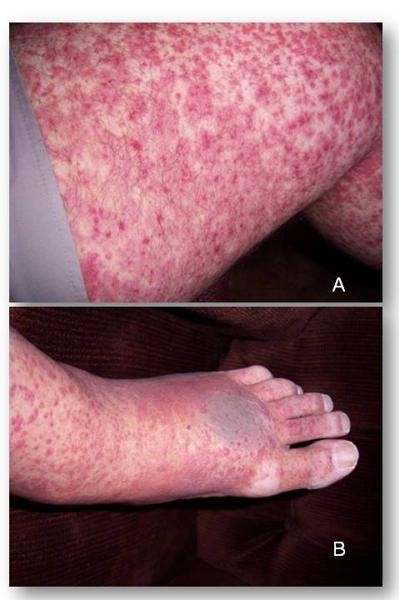
**A 56 year old man with prior history of hypertension, diabetes mellitus presented with a 4 week-duration skin eruption over the lower extremities (Figure 1A and 1B)**. This was accompanied by crampy lower abdominal pain and hematuria. Punch biopsy of involved skin demonstrated leukocytoclastic vasculitis, accompanied by deposition of IgA on vascular walls on direct immunofluorescent staining.

A 56 year old man with prior history of hypertension and adult onset diabetes mellitus presented with a skin eruption over the lower extremities of several weeks duration (appearance of the eruption is shown in Figures 1A and 1B). This was accompanied by intermittent, crampy lower abdominal pain and hematuria. The patient denied fever, weight loss, diaphoresis, or arthralgia. He denied a history of a preceding upper respiratory infection. Examination of the patient demonstrated a palpable purpuric eruption over the lower extremities (Figure 1 A and 1B). Urinalysis revealed proteinuria as well as microscopic hematuria. His serum IgA levels was elevated (787 mg/dL with a normal range of 70-400 mg/dL), while tests for lupus, vasculitis and hepatitis were negative (Table 1). A skin biopsy showed an inflammatory infiltrate around superficial blood vessels with associated nuclear dust and focal fibrinoid necrosis, consistent with leukocytoclastic vasculitis. This was accompanied by deposition of IgA on vascular walls seen on direct immunofluorescent staining, a pathognomonic feature of HSP.

**Table 1:** Summary Clinical and Laboratory reviews

Case	Case 1	Case 2
Age (years)	56	57

Respiratory infection	No	No

Eruption	Palpable purpuric rash	Blistering rash

Abdominal pain	Crampy abdominal pain	No

Arthritis/Arthralgia	No	No

Proteinuria g/24 hours	2.7	3.7

Microscopic hematuria	Yes	Yes

White cell count	8.0 × 10^3 ^cells/mm^3^	13.8 × 10^3 ^cells/mm^3^

Platelet count	164 × 10^3 ^cells/mm^3^	294 × 10^3 ^cells/mm^3^

PT/INR	Normal	Normal

ESR (mm/hour)	19	17

CRP(0-9.9) mg/L	11.1	220

Serum IgA mg/dL	787	272

C3/C4	Normal	Normal

Autoantibodies	Negative	Negative

Skin biopsy with immunofluorescence	IgA deposition LCV	IgA deposition LCV

Renal biopsy with immunofluorescence	N/A	IgA deposition

Treatment	Colchicine and Steroid	Colchicine and Steroid

LCV = leukocytoclastic vasculitis

#### Case 2

**Figure 2:**
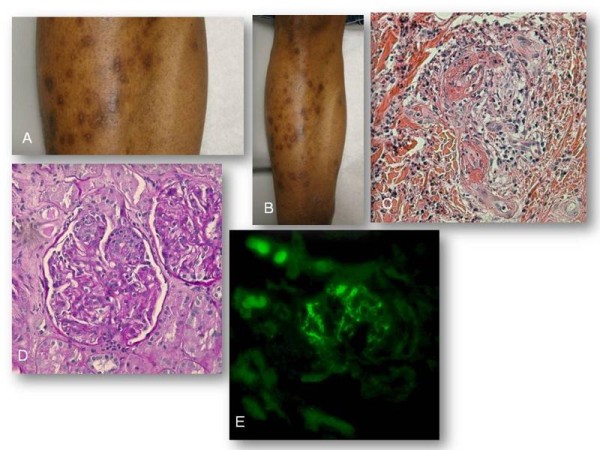
**Examination revealed edema and a vesiculobullous rash in both hands and feet that showed varied degrees of healing (Figure 2A and B)**. Skin biopsy revealed the presence of a perivascular inflammatory infiltrate in the superficial dermal blood vessels, with nuclear dust and focal fibrinoid necrosis, consistent with leukocytoclastic vasculitis (Figure 2C; Hematoxylin and eosin stain-40x objective), accompanied by the deposition of IgA on vascular walls detected by direct immunofluorescent staining. Renal biopsy revealed focal segmental endocapillary proliferation (Figure 2D; Periodic acid- Schiff stain-40x objective) with positive immunofluorescence testing for mesangial IgA (Figure 2E; Immunoflurescence stain-40x objective)

A 57 year old African American man with prior history of cigarette smoking and alcohol abuse, hypertension, coronary artery disease, and hyperlipidemia presented with one week duration of blistering rash on both ankles ascending to his groin area, and involving his hands. This was associated with swelling in both hands and feet without any abdominal pain. He denied any history of antecedent upper respiratory tract infection. Examination revealed edema and a vesiculobullous rash in both hands and feet that showed varied degrees of healing (Figure 2A and 2B). Skin culture growth Stenotrophomonas maltophilia. Urinalysis revealed microscopic hematuria and proteinuria. The spot urine protein-creatinine ratio was 3.7 which confirmed the diagnosis of nephrotic syndrome. Other laboratory results were unremarkable, including hepatitis testing and serologies, with the exception of an elevated CRP level of 220 mg/dL (normal: 0-9.9 mg/dL: Table 1). Skin biopsy revealed the presence of a perivascular inflammatory infiltrate in the superficial dermal blood vessels, with nuclear dust and focal fibrinoid necrosis, consistent with leukocytoclastic vasculitis(LCV) (Figure 2C), accompanied by the deposition of IgA on vascular walls detected by direct immunofluorescent staining, a pathognomonic feature of HSP. Renal biopsy revealed focal segmental endocapillary proliferation (Figure 2D) with positive immunofluorescent staining for mesangial IgA (Figure 2E).

### Pathogenesis

**Figure 3:**
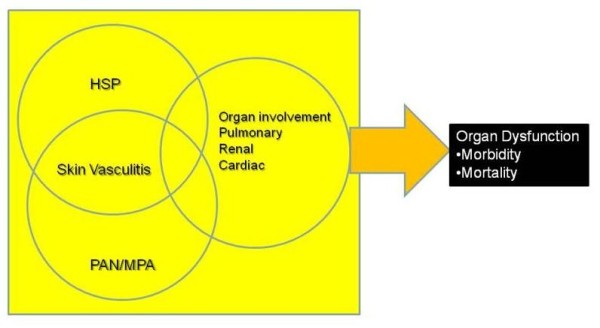
**In some HSP cases reported in the literature, overlap with microscopic polyangiitis (MPA) or Polyarteritisa Nodosa (PAN)-like disease have been reported, resulting in a plethora of severe renal and pulmonary manifestations**.

Henoch-Schonlein Purpura (HSP) is a small vessel vasculitis associated with immunoglobulin A(IgA) complex deposition [2,4]. The immune complexes are composed of IgA1 and IgA2 but only IgA1 is found in the inflammatory infiltrate of the disease [5]. Polymorphonuclear leukocytes are recruited by chemotactic factors and cause inflammation and necrosis of vessel walls (focal fibrinoid necrosis) with occasional thrombosis, and with associated red blood cell extravasation, consistent with a form of leukocytoclastic vasculitis [6]. Skin biopsy reveals polymorphonuclear cells or cell fragments around small dermal blood vessels. Immune complexes containing IgA and C3 have been found in skin, intestinal mucosa, joints, and kidneys which are the major organ sites involved in HSP [6]. In some cases reported in the literature, overlap with polyangiitis or polyarteritisa nodosa-like disease have been reported, resulting in a plethora of severe renal and pulmonary manifestations (Figure 3). Although these cases are anecdotal, their concurrence in a given patient suggests common pathways of immune complex-mediated vasculitis and resultant tissue injury.

### Etiology

The etiology of HSP remains unknown. Various antigenic stimuli have been proposed to trigger this pathology, including a broad spectrum of infectious agents such as infections due to group A Streptococcus, Methicillin-resistant Staphylococcus aureus, Helicobactor pylori, Parvovirus B19, Hepatitis B, Human Immunodeficiency Virus, Stenotrophomonas maltophilia (as seen in the second patient reported in this study). Other etiologies proposed including allergens such as drugs, tumor antigens associated with malignancy and certain autoimmune diseases [2,7-11]. Ninety percent of cases occur in children with a favorable outcome as stated earlier, suggesting either unique infectious pathogens common in childhood or other unknown genetic/molecular factors.

### Clinical Features

Adult-onset HSP been described [4,8,12-20], (though 90% of cases still occur in children), with only 3.4 to 14.3 cases per million reported in the adult population [4]. The diagnostic criteria for HSP are shown in Table 2. We used PubMed to review the English literature for adult-onset HSP cases and the salient aspects of selected cases along with our patients are shown in Table 3. The clinical tetrad of presentations may be in any sequence, with upper respiratory infection prior to the onset of symptoms in about 36.4% [3]. A retrospective review of 250 adult patients with the disease reported on the following clinical findings [19]: a purpuric rash occurred in 96%, arthritis in 61%, GI disease in 48% and renal disease in 32% cases (99% of these cases demonstrating proteinuria and 93% hematuria).

**Table 2:** Diagnostic criteria for Henoch-Schonlein Purpura

The European League Against Rheumatism and Paediatric Rheumatology European Society criteria --2006	
***Mandatory criterion***: Palpable purpura with lower limb predominance	
***Plus at least one of the following criteria:***	
1.	Diffuse abdominal pain
2.	IgA deposition in any biopsy
3.	Arthritis/arthralgia
4.	Renal involvement (hematuria and/or proteinuria)

**American College of Rheumatology criteria--1990**	

***Two or more of the following criteria are needed***	
1.	Age 20 years or less at disease onset
2.	Palpable purpura
3.	Acute abdominal pain with gastrointestinal bleeding
4.	Biopsy showing granulocytes in the walls of small arterioles or venules in superficial layers of skin

**Table 3:** Selected reports of HSP in adult patients

Patient (report)	Age/gender	Clinical/Labs	Histological diagnosis	Treatment	Outcome
1	24/M	Purpuric rash, arthritis, abdominal pain, Hematuria and proteinuria	Skin: LCV with IgA deposition Mesangial IgA deposition	Glucocorticoids Cyclophosphamide	Remission

2	68/M	Abdominal pain with diarrhea, purpuric rash, elevated sedimentation rate	Skin: LCV with IgA deposition	None	Spontaneous remission

3	77/M	Abdominal pain with diarrhea purpuric rash, hematuria, elevated IgA level and sedimentation rate	Skin: LCV with IgA deposition	Glucocorticoids Cyclophosphamide	Remission

4	69/M	Pustular rash, abdominal pain myocardial infarction	Endocapillary proliferative nephritis with IgA deposition subendocardial LCV	Glucocorticoids	Deceased

5	20/M,76/F, 67/F	Purpuric rash, arthralgia, hematuria hemoptysis, hypoxia, bilateral infiltrate	Skin: LCV with IgA deposition pulmonary interstitial fibrosis	Glucocorticoids	Remission

6	20/F	Purpuric rash, arthralgia, hematuria, proteinuria, seizure	Skin:LCV EEG: Transient focal abnormality MRI: normal	Glucocorticoids Dilantin	Remission

7*	56/M	Purpuric rash, crampy abdominal pain Hematuria, proteinuria, elevated IgA level	Skin: LCV	Colchicine	Remission

8*	57/M	Blistering rash, hematuria, Nephrotic syndrome	Skin: LCV Endocapillary proliferative nephritis mesangial IgA deposition	Glucocorticoids Colchicine	Partial remission

LCV: Leukocytoclastic vasculitis; M = Male; F = Female

* Cases described in this report

The skin is the most commonly involved site, often starting with an erythematous, macular, or urticarial-type eruption. The lesions then coalesce and evolve into the typical ecchymoses and/or palpable purpura. The lesions tend to appear in crops, with a symmetrical distribution over gravity/pressure-dependent areas such as lower limbs, trunk and upper extremities and frequently accompanied by edema on both extremities. Blistering and hemorrhagic necrotic skin lesions occur in 35% [4,19].

Arthritis/arthralgia, the second most common manifestation, occurs in 61% of cases. It usually transient or migratory, oligoarticular and non-deforming, but often associated with periarticular swelling and tenderness without effusion [4,19]

Gastrointestinal involvement is a typical feature of HSP [19,21-23] and occurs in about 48% of cases. It is caused by submucosal hemorrhage and edema or by vasculitis. The most common presentation is colicky pain or bleeding from an ulcer usually in the second part of duodenum or ileum and/or the rectum. Gastrointestinal symptoms usually develop about one week after the onset of the rash. In about 8% of cases, it can be the first clinical presentation. In this situation, endoscopy and/or colonoscopy may help establish the diagnosis [4,19]. Intussusception may occur in elderly, often presenting as an acute abdominal emergency and may require urgent radiological evaluation for the diagnosis [22]. HSP also has been linked with primary biliary cirrhosis and transient abnormalities of liver function tests. As in our patients, an elevated IgA level occurs in about 60% and may point to the diagnosis, when the clinician is faced with an unusual lower extremity eruption in the setting of abdominal pain.

Renal impairment, the most serious complication, ranges from microscopic hematuria to a full blown nephrotic syndrome [19]. It is detected in 45-85%, with a risk of progression to renal insufficiency in 30% of adult-onset HSP. It is usually detected within two months of the eruption, but sometimes may manifest as late as six months after initial onset of the disease [3]. The most frequent pathology observed is a mesangial or endocapillary proliferative glomerulonephritis [19]. Some studies have suggested that certain genetic polymorphorphisms, such as those involving cytokine genes (IL1 receptor antagonist, IL8 or IL1 beta) have been associated with increased risk of renal involvement [24-26]. Age of onset, the presence of renal impairment and hematuria at the onset, abdominal pain as an initial presentation, persistent eruption, renal pathology with fibrinoid necrosis and the number of sclerotic glomeruli are significant predictors of renal disease [3,19,27].

### Other Complications

Unusual presentations include pulmonary involvement [28-30], scrotal pain [31], central, peripheral nervous system involvement and seizure [32-34] as well as cardiac involvement [35-37]. Pulmonary involvement can manifest as diffuse alveolar hemorrhage and occasionally as usual interstitial pneumonia or interstitial fibrosis. There is an association between HSP and malignancy [7,9,38], most commonly associated with solid tumors. An evaluation for occult malignancy may therefore reasonable in adults (especially those above 40 years of age) diagnosed with HSP.

### Diagnosis

There are two criteria proposed by American College of Rheumatology [39] and the new criteria by European League Against Rheumatism (EuLAR) and Pediatric Rheumatology Society (PReS) [40] as listed in Table 2. The diagnosis is usually based on clinical presentation with tissue biopsy demonstrating leukocytoclastic vasculitis associated with IgA deposition (by immunofluorescence). Skin biopsy should be obtained from the lesion less than 24 hours old and typically shows the classical leukocytoclastic vasculitis in postcapillary venule with IgA deposition. Renal biopsy should be performed in case of uncertain diagnosis or severe renal impairment such as nephrotic syndrome. Endoscopy and/or colonoscopy play a major role in helping diagnosis of the patients with the gastrointestinal involvement as their initial presentation.

No specific test is diagnostic for HSP. Elevated serum IgA levels have been associated with HSP in about 60% of cases [19]. Urine analysis can vary from microscopic hematuria to nephritic-syndrome range proteinuria. Coagulation studies and the platelet count are usually normal. Inflammatory markers such as sedimentation rate (ESR) and C reactive protein (CRP) levels are often elevated. Serum level of insulin like growth factor (IGF) and IGF binding protein 3 have been proposed as a marker for determination of renal involvement as well as the terminal complement complex (SC5b-9) level as a surrogate for disease activity in HSP [41]. These need independent confirmation.

### Treatment

Most cases of LCV are self-limited and may require little or no intervention. In mild cases a nonsteroidal anti-inflammatory agent may suffice. Colchicine is the treatment of choice when the skin lesions are severe [42]. Colchicine inhibits polymorphonuclear leukocyte chemotaxis by inhibiting spindle formation, blocking lysosome formation and stabilizing the lysosome membranes. The suppressive effect of colchicine on the inflammatory pathway may explain its efficacy on the skin lesions. The sulfone, Dapsone, has also been used to treat the vasculitic component of HSP; it has antioxidant scavenger effects and may suppress the generation of toxic free radicals in neutrophils. It also inhibits the synthesis of IgG and IgA antibodies and prostaglandin D_2 _[43]. Glucocorticoids (such as prednisone at a dosage of 1-2 mg/kg daily) have been used to treat gastrointestinal symptoms successfully [44].

Aggressive therapy with corticosteroids or cyclophosphamide has not been shown to be beneficial in reversing the renal disease except among patients with the crescentic form of nephritis. Plasmapheresis and immunoglobulin have been used in refractory combination therapy. These treatment options are summarized in Table 4[43,45-49].

**Table 4:** A summary of treatment options for Henoch-Schonlein Purpura

Medication	Indication
Acetaminophen, NSAID	Mild eruption, arthritis

Colchicine	Severe or recurrent skin disease

Oral glucocorticoids	Severe eruption, cutaneous edema, severe colicky abdominal pain, scrotal and testicular involvement

Parenteral glucocorticoids	Same as oral; unable to tolerate oral medications

High dose parenteral pulse glucocorticoid	Nephrotic range proteinuria

High dose IV pulse glucocorticoids combined with other forms of immunosuppression (such as cyclophosphamide)	Rapidly progressive glomerulonephritis Hemorrhagic involvement of lungs or brain

Plasmapheresis and/or IGIV	Refractory to combination therapy Massive hemorrhage in gastrointestinal or other organs

IV = intravenous; IGIV = intravenous immunoglobulin

## Conclusion

HSP is a heterogeneous disorder manifesting in adults with palpable purpura/skin vasculitis, hematuria and proteinuria, often with preserved renal function. The diagnosis can be easily missed: A high degree of suspicion and requesting immuno-fluorescence studies in suspected cases are mandatory to establishing the diagnosis. Skin biopsy and immunofluorescence confirm the presence of LCV with IgA deposition which is the pathognomonic finding in HSP. Colchicine is a treatment of choice for severe or recurrent LCV. Adults with HSP carry a different prognosis, and the development of hematuria may be a harbinger for more serious complications such as nephritic or nephrotic syndrome. Malignancy is common in adult onset HSP and work up should be done to exclude this possibility.

## Competing interests

The authors declare that they have no competing interests.

## Authors' contributions

WJ carried out the literature review, case report description, and clinical presentations

YM carried out the pathology sections and prepared for pathology slides and legends

HS participated in clinical presentation

GY participated in pathology sections advisors

DC assisted in review

GK provided the material and patient data, organized the manuscript, edited figures, assisted in discussion, generated references and participated in the editing and final approval of the manuscript

All authors read and approved the final manuscripts.
